# The Scientific American

**Published:** 1877-02

**Authors:** 


### BIBLIOGRAPHICAL.

*The Scientific American— What Pays.*—It pays every Manufacturer, Merchant, Mechanic, Inventor, Farmer, or Professional man, to keep informed on all the improvements and discoveries of the age.

It pays the head of every family to introduce into his household a newspaper that is instructive, one that fosters a taste for investigation, and promotes thought and encourages discussion among the members.

The Scientific American which has been published weekly for the last thirty-one years, does this, to an extent beyond that of any other publication ; in fact it is the only weekly paper published in the United States, devoted to Manufactures, Mechanics, Inventions and New Discoveries in the Arts and Sciences.

Every number is profusely illustrated and its contents embrace the latest and most interesting information pertaining to the industrial, Mechanical, and Scientific Progress of the World; Descriptions, with Beautiful Engravings of New Inventions, New Implements, New Processes, and Improved Industries of all kinds ; Useful Notes, Recipes, Suggestions and Advice by Practical Writers, for Workmen and Employers, in all the various arts, forming a complete repertory of New Inventions and Discoveries ; containing a weekly record, not only of the progress of the Industrial Arts in our own country, but also of al New Discoveries and Inventions in every branch of Engineer-l ing, Mechanics, and Science abroad.

The Scientific American has been the foremost of all industrial publications for the past thirty-one years. It is the oldest, largest, cheapest, and the best weekly illustrated paper devoted to Engineering, Mechanics, Chemistry, New Inventions, Science and Industrial Progress, published in the world.

The practical Recipes are well worth ten times the subscription price, and for the shop and house will save many times the cost of subscription.

Merchants, Farmers, Mechanics, Engineers, Inventors, Manufacturers, Chemists, Lovers of Science, and People of all Professions, will find the Scientific American useful to them. It should have a place in every Family, Library, Study, Office, and Counting Room; in every Reading Room, College and School. A new volume commences January 1st, 1877.

A year's numbers contain 832 pages and several hundred engravings. Thousands of volumes are preserved for binding and reference. Terms, $3.20 a year by mail, including postage; Discount to Clubs. Special circulars, giving Club rates, sent free Single copies mailed on receipt of 10 cents. May be had of all News Dealers.

In connection with the Scientific American, Messers. Munn & Co. are Solicitors of American and Foreign Patents, and have the largest establishment in the world. More than fifty thousand applications have been made for patents through their agency.

Patents are obtained on the best terms, Models of New Inventions and sketches examined, and advice free. A special notice is made in the Scientific American of all Inventions Patented through this Agency, with the name and residence of the Patentee. Patents are often sold in part or whole, to persons attracted to the invention by such notice. A Pamphlet, containing full directions for obtaining Patents sent free. The Scientific American Reference Book, a volume bound in cloth and gilt, containing the Patent Laws, Census of the U. S., and 142 Engravings of mechanical movements. Price 25 Cents.

Address for the paper, or concerning Patents, Munn & Co., 37 Park Row, New York. Branch Office, Cor. F. and 7th Sts., Washington D. C

